# The relationship between psychological *Suzhi* and social anxiety among Chinese adolescents: the mediating role of self-esteem and sense of security

**DOI:** 10.1186/s13034-018-0255-y

**Published:** 2018-12-13

**Authors:** Zhaoxia Pan, Dajun Zhang, Tianqiang Hu, Yangu Pan

**Affiliations:** 1 Faculty of Psychology, Research Center for Mental Health Education, Southwest University, Chongqing, 400715 China; 2 Faculty of Education Science, Kaili University, Kaili, 556001 China; 3 Research Institute of Social Development, Southwestern University of Finance and Economics, Chengdu, 611130 China

**Keywords:** Adolescents, Psychological *suzhi*, Self-esteem, Sense of security, Social anxiety

## Abstract

**Background:**

High incidence and morbidity rates are found among adolescents with social anxiety disorder, a severe and harmful form of social phobia. Extensive research has been conducted to uncover the underlying psychological factors associated with the development and continuation of this disorder. Previous research has focused on single individual difference variables such as personality, cognition, or emotion; thus, the effect of an individual’s full psychological profile on social anxiety has rarely been studied. Psychological *suzhi* is a comprehensive psychological quality that has been promoted in Chinese quality-oriented education. This research aimed to explore how psychological *suzhi* affects Chinese adolescents’ social anxiety.

**Methods:**

A cross-sectional survey study was carried out among 1459 middle school students (683 boys and 776 girls) from various middle schools in seven provinces of China. Psychological s*uzhi*, self-esteem, sense of security, and social anxiety were measured via four self-reported questionnaires: the Brief Psychological *Suzhi* Questionnaire for middle school students, the Chinese version of the Rosenberg Self-Esteem Scale, the Security Questionnaire, and the Social Avoidance and Distress Scale.

**Results:**

Analyses showed that psychological *suzhi* is positively related to self-esteem and sense of security, and it is negatively correlated with social anxiety. The results also revealed that self-esteem partially mediates the relationship between adolescents’ psychological *suzhi* and social anxiety, with self-esteem and sense of security serving as chain mediators in the relationship between psychological *suzhi* and social anxiety.

**Conclusions:**

Results highlight that psychological *suzhi* is a protective factor against social anxiety. It can directly protect adolescents from social anxiety, and it also can protect them through affecting their self-esteem and sense of security. These results are discussed from the viewpoints of school leaders, psychology teachers, and school counsellors, who provide support to students to improve their social functioning within the school context. The findings of this study may provide new perspectives regarding the prevention and treatment of social anxiety.

## Background

Psychological *suzhi* is an endogenous Chinese psychological concept that has been promoted within the background of Chinese quality-oriented education [1] and has subsequently roused the interest of many Chinese psychologists [2]. The concept of psychological *suzhi* became more widely known following the publication of an internationally authoritative reference book, *The Handbook of Positive Psychology in Schools* [3], wherein it was recognised as a concept of positive psychology. Psychological *suzhi* is defined as a fundamental, stable, and implicit mental quality that forms under the influence of inborn conditions, the environment, and one’s education. It is closely and positively associated with an individuals’ adaptive, developmental, and creative behaviors [1, 4]. Psychological *suzhi* is a comprehensive mental quality that comprises three elements: cognitive quality, individuality, and adaptability. Cognitive quality is the most fundamental component, which directly involves individuals’ cognitive process. Individuality is reflected through one’s action towards that reality and plays a motivating and moderating function during cognition. Finally, adaptability refers to the ability to make oneself be in harmony with the environment; it is the functional component of psychological *suzhi* that reflects the other two components’ states [4]. It is a weighty component of students’ quality, which Chinese quality education is designed to cultivate. To explore the positive function of this important quality component, a series of studies concerning the relationship between psychological *suzhi* and mental health have been conducted and they have found that psychological *suzhi* negatively predicts depression [5]. However, it has been positively associated with life satisfaction [6], subjective well-being [7], and positive emotions [8]. Based on the results of the above studies, researchers have constructed a psychological *suzhi* and mental health relationship model, and proposed that psychological *suzhi* is an endogenous factor that affects mental health [9].

Social anxiety is a negative indicator of mental health. It begins at puberty and is most common among teenagers [10]. Related research has also revealed that many members of this demographic group have at least moderate impairment in their socio-emotional functioning [11], academic achievement [12], quality of life [13], areas of friendship [14], and even emerging adult relationship quality [15]. These impairments may result in increased likelihood of engaging in cigarette smoking [16] and drinking alcohol [17]. Given the high prevalence of social anxiety and its harmful nature among middle school students, extensive research has been conducted to uncover the underlying psychological factors associated with the development and maintenance of this condition. Such research has revealed that personality [18]; irrational social, cognitive [19], and behavioural patterns [20, 21]; and information processing biases [22] are important factors that can influence the development of social anxiety. Further, Chinese adolescents’ psychological *suzhi* can also influence their social anxiety levels. Liu et al. [23] discovered that psychological *suzhi* was a protective factor against social anxiety. However, a thorough examination of it as a comprehensive psychological quality—i.e. how it protects individual against social anxiety—was lacking. Therefore, in order to further reveal the relationship between psychological *suzhi* and mental health, and to reveal how multiple variables interact to influence the symptoms of social anxiety, it is necessary to explore how the mechanism of psychological *suzhi* affects social anxiety. Understanding this mechanism would provide a basis for the effective prevention and scientific control of social anxiety.

Cognitive and behavioural theories of social anxiety emphasise the influence of low self-evaluation on individuals’ development of social anxiety [24]. Indeed, some empirical studies have verified the negative relationship between self-esteem and social anxiety [25], while others have determined that psychological *suzhi* is a powerful motivator of self-esteem [7, 23]. Thus, individuals with high psychological *suzhi* have high levels of self-esteem and, in turn, low levels of social anxiety. Therefore, self-esteem may play a mediating role between psychological *suzhi* and social anxiety.

Sense of security is defined as an individual’s physical or mental feelings concerning the level of danger and risk in their surroundings, as well as their sense of power or powerlessness to address any such dangers. It is mainly manifested in terms of interpersonal security and feelings of control [26]. Sense of security is one of the most important determinants of mental health and is considered a basic human need [27]. Further, empirical research has shown that it is an important factor in the development of social anxiety [28]. However, with regard to the relationship between self-esteem and sense of security, there is controversy concerning the direction of specific predictions. Some researchers, in accordance with Maslow’s hierarchy of needs, have proposed that security is a basic need; only when security needs are met can an individual work toward the fulfilment of needed self-esteem. However, other researchers insist that individuals with low self-esteem are unable to develop feelings of security because they lack confidence, and that high self-esteem is more likely to produce a sense of security [29]. Although we believe that there are merits to both arguments in the above debate, one definition of sense of security must be chosen in order to clarify its relationship with self-esteem. Given the measures used in the current study, we adopt the latter viewpoint in terms of our understanding and definition of this construct. Therefore, this study assumes that self-esteem predicts sense of security, which, in turn, predicts social anxiety. In other words, sense of security is assumed to act as a mediating variable between self-esteem and social anxiety. In this context, psychological *suzhi* positively predicts self-esteem, which affects an individuals’ sense of security, and sense of security negatively predicts social anxiety. Thus, self-esteem and sense of security may serve as chain mediators in the relationship between psychological *suzhi* and social anxiety.

Although there is currently no research demonstrating the close relationship between psychological *suzhi* and sense of security, some explanations concerning this relationship have been offered in other studies. Zhang [4] proposed that personality elements that have adaptive and health functions are the basic components of psychological *suzhi*, and that personality is also closely related to psychological *suzhi*. Meanwhile, Xie et al. [30] found that psychological *suzhi* is positively related to extraversion and negatively related to neuroticism. Research on the relationship between personality and sense of security has also indicated that personality can predict sense of security; specifically, sense of security is positively and negatively predicted by extraversion and neuroticism, respectively [31]. In this context, psychological *suzhi* may be positively correlated with sense of security, and sense of security may play a mediating role in the relationship between psychological *suzhi* and social anxiety.

Based on the relationships described above, we can know that: first, previous studies on the factors that influence social anxiety have generally examined one or several separate individual difference variables such as personality, cognition, or emotion [32]. The effect of an individual’s full psychological profile on social anxiety has rarely been studied. Consequently, in this study we investigated the influence of the Chinese comprehensive psychological variables, psychological *suzhi*, on social anxiety to reveal the factors influencing social anxiety among Chinese adolescents. Second, the intrinsic mechanism of this relationship was unknown; therefore, based on the cognitive and behavioural theories of social anxiety and the theory of the sense of security, we investigated the roles of self-esteem and a sense of security as mediators in the relationship between psychological *suzhi* and social anxiety. This research can provide valuable references for prevention of social anxiety and its related interventions.

**Fig. 1:**
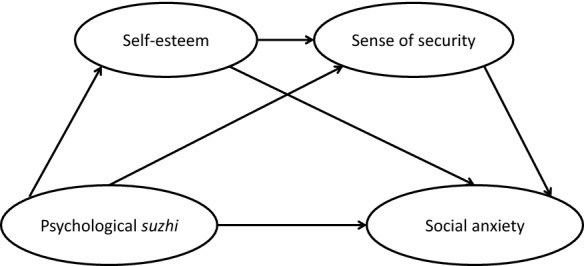
Model of the hypothesised mediating roles of self-esteem and sense of security in the relationship between psychological *suzhi* and social anxiety

Our specific hypotheses were as follows: (1) psychological *suzhi* is positively related to self-esteem and sense of security, but it is negatively related to social anxiety; and (2) self-esteem and sense of security mediate the relationship between psychological *suzhi* and social anxiety. A detailed model of the hypothesised mediating role of self-esteem and sense of security in the relationship between psychological *suzhi* and social anxiety is presented in Fig. 1.

## Methods

### Participants and sample

The current study is part of the national, normative measurement of psychological *suzhi* among Chinese middle school students. This national sampling was conducted from October to December 2016. The whole group stratified random sampling method was used to extract the subjects. The inclusion criteria were: (1) being a full-time, middle school student; and (2) being between the ages of 11 and 18 years. Because this was a normative measurement of middle school students’ psychological *suzhi*, there were no exclusion criteria. This study was approved by the research ethics committee of the author’s institution. Written consent was obtained from the heads of participating middle schools and the participants’ parents, and the student participants provided their oral assent.

In this study, 34 classes of students from junior and senior middle schools in the Beijing, Guangdong, Zhejiang, Henan, Jiangxi, Sichuan, and Chongqing provinces were selected to complete a self-administered questionnaire. A total of 1587 students were approached to participate in this study. Under the guidance of a trained investigator, the participants were given 40 min to complete a series of self-report questionnaires during normal class time. They returned their anonymous questionnaires to the researcher upon completion. After completing the questionnaire, each participant received 5 RMB as compensation. Ultimately, 1459 valid questionnaires were recovered, with an effective recovery rate of 91.9%. The participants were representative of the total sample in terms of age, gender, and grade.

### Measures

#### Psychological *suzhi*

To measure psychological *suzhi*, we used the Brief Psychological *Suzhi* Questionnaire for middle school students (BPSQM) [33], which is specifically designed to measure middle school students’ psychological *suzhi* in a Chinese environment. It contains 24 items and assesses three dimensions of psychological *suzhi*: cognitive quality, individuality, and adaptability. The items are presented on a 5-point Likert scale, with responses ranging from 1 (*not at all true for me*) to 5 (*extremely true for me*). Consequently, overall scores range from 24 to 120, with higher scores reflecting higher psychological *suzhi*. The brief BPSQM was validated using a large sample of Chinese students (*N *= 2549), and its psychometric properties were found to support a bi-factor structure. Additionally, the total scale was found to have excellent internal consistency (Cronbach’s *α *= .91), and the subscales were all determined to have acceptable internal consistency (*α *> .76) [33]. In this study, Cronbach’s alpha for the total scale was .94 and ranged between .84 and .87 for the three subscales.

#### Self-esteem

Self-esteem was assessed using the Chinese version of the Self-Esteem Scale (SES) [34]. The SES contains 10 items presented using a 4-point Likert scale for which the responses range from 1 (*not at all true for me*) to 4 (*extremely true for me*). Overall scores ranged between 10 and 40. The Chinese version of the SES has been widely used among the Chinese population and has been demonstrated to be a reliable and valid measure. Based on the findings of a previous study, we chose to omit one item (item 8), as it has been found to have low factor loadings within a Chinese context [35]. Consequently, Cronbach’s alpha for the final scale was .88 in the current study.

#### Sense of security

Sense of security was assessed using the Security Questionnaire (SQ) [26], which contains 16 items divided into two subscales: interpersonal security (eight items) and certainty in control (eight items). The interpersonal security subscale assesses feelings of security during interpersonal communication, while the certainty in control subscale assesses sense of control over life and life uncertainty. Items are presented on a 5-point Likert scale, with responses ranging from 1 (*extremely true for me*) to 5 (*not at all true for me*). Further, overall scores range from 16 to 90, with higher scores reflecting a higher sense of security. In this study, Cronbach’s alpha was .88 for the total scale, .78 for the interpersonal security subscale, and .83 for the certainty in control subscale.

#### Social anxiety

Social anxiety was assessed using the Social Avoidance and Distress Scale (SADS) [36]. The SADS contains 28 items that comprise two subscales: social avoidance (14 items) and social distress (14 items). The social avoidance subscale assesses avoidance behaviour and the desire to avoid situations that involve interactions, whereas the social distress subscale assesses the degree of negative emotions experienced during social interactions. Participants provide a ‘yes’ or ‘no’ answer to each item. The Chinese version of the SADS has been found to exhibit acceptable reliability and validity in adolescent studies [37]. In the current study, Cronbach’s alpha was .87 for the total scale, .77 for the social avoidance subscale, and .80 for the social distress subscale.

### Data analysis

Data were analysed using SPSS 19.0 and MPlus 7.0 [38]. The first purpose of this study was to investigate the correlation between psychological *suzhi*, self-esteem, sense of security, and social anxiety. To this end, descriptive statistics and Pearson’s correlational analyses were conducted using SPSS 19.0. The second purpose was to examine the mediation model, so a path analysis using structural equation modelling was used to test the direct and indirect effects of psychological *suzhi* on social anxiety. The model included four latent variables (psychological *suzhi*, self-esteem, sense of security, and social anxiety) that were made up of 12 parcels to reduce model complexity [39, 40]; the average scores for each parcel were used as indicators in the model. The model included a direct effect of psychological *suzhi* on social anxiety and three indirect effects through self-esteem and sense of security: psychological *suzhi *→ self-esteem → social anxiety; psychological *suzhi *→ sense of security → social anxiety; and psychological *suzhi *→ self-esteem → sense of security → social anxiety. Missing data were estimated using full information maximum likelihood estimation, and robust maximum likelihood estimation was used to account for non-normality. Meanwhile, standardized regression coefficients (β) were presented to quantify the strength of association between pairs of variables. The indirect effects of the model were checked using bootstrapping procedures [39], and model fit was evaluated using several common fit indices: CFI, TLI, RMSEA, and SRMR. The following were considered indices of good fit: CFI > .90, TLI > .90, RMSEA < .08, and SRMR < .08 [41].

## Results

### Sample descriptives

Table 1 displays the descriptive statistics for the sample. The 1459 included participants had a mean age of 14.83 years (SD = 1.83 years). Among them, 684 (46.9%) were boys, and 775 (53.1%) were girls. Concerning grade, 241 (16.5%), 216 (14.8%), 218 (14.9%), 260 (17.8%), 285 (19.5%), and 239 (16.4%) were in seventh, eighth, ninth, tenth, eleventh, and twelfth grades, respectively. Regarding province, 218 (14.9%), 104 (7.1%), 98 (6.7%), 172 (11.8%), 580 (39.8%), 105 (7.2%), and 182 (12.5%) were from Beijing, Zhejiang, Guangdong, Henan, Jiangxi, Shanxi, and Sichuan, respectively. Moreover, the participants were almost entirely of Han ethnicity (98.9%), with the remainder being from ethnic minorities.

**Table 1:** Sample descriptive statistics

Variable	Category	Frequency	Percent
Gender	Male	684	46.9
	Female	775	53.1
Grade	7th	241	16.5
	8th	216	14.8
	9th	218	14.9
	10th	260	17.8
	11th	285	19.5
	12th	239	16.4
Ethnicity	Han ethnicity	1443	98.9
	Ethnic minorities	16	1.1
Province	Beijing	218	14.9
	Zhejiang	104	7.1
	Guangdong	98	6.7
	Henan	172	11.8
	Jiangxu	580	39.8
	Shanxi	105	7.2
	Sichuan	182	12.5

### Preliminary analyses

We determined the means, standard deviations, and bivariate correlations of all the variables, as shown in Table 1. Results indicated that psychological *suzhi* was positively correlated with self-esteem and sense of security (*r *= .29–.52, *p *< .01), and it was negatively correlated with social anxiety (*r* = − .34, *p* < .01). Analyses of the potential covariates indicated that gender was positively related to sense of security and social anxiety (*r* = .09–.14, *p* < .01), and it was negatively related to self-esteem (*r* = − .12, *p* < .01). In addition, grade was positively related to sense of security (*r* = .06, *p* < .01) and negatively related to psychological *suzhi* (*r* = − .15, *p* < .01). Thus, gender and grade were included as covariates in subsequent analyses.

### Measurement model

A confirmatory factor analysis was used to test the fit of the measurement model. Here, the abovementioned four latent variables (psychological *suzhi*, self-esteem, sense of security, and social anxiety), with 12 parcels as indicators, comprised the measurement model. Results indicated that the data fit the model well: χ^2^ (47) = 272.591; CFI = .963; TLI = .947; RMSEA = .057 (90% CI [.051, .064]); SRMR = .036. Further, all factor loadings on the latent variables were significant (*p *< .01), indicating that the latent factors were well represented by their respective indicators.

### Structural model

**Fig. 2:**
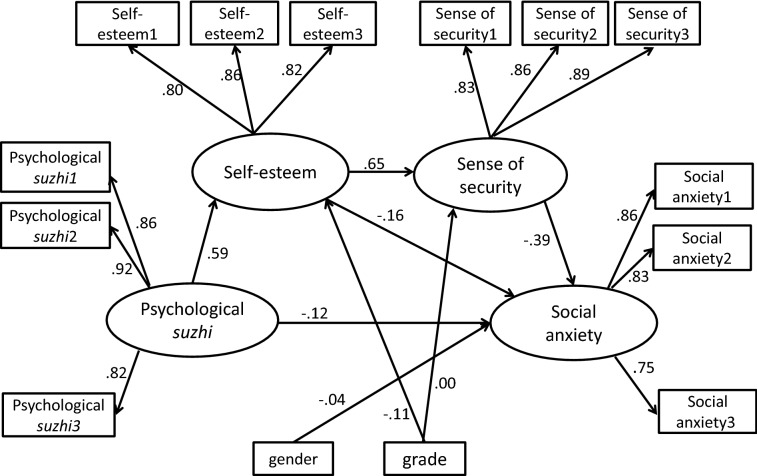
Structural equation model of the proposed relationships between psychological *suzhi*, self-esteem, sense of security and social anxiety

As shown in Fig. 2 and Table 2, after controlling for gender and grade, the structural model examining the relationship between psychological *suzhi*, self-esteem, sense of security, and social anxiety fit the data well: ⎟^2^(63) = 536.334, *p *< .001; CFI = .956; TLI = .937; RMSEA = .072 (90% CI = [.066, .077]); SRMR = .040. Analyses of the total indirect effects indicated that self-esteem and sense of security partially mediated the relationship between psychological *suzhi* and social anxiety (^*®*^= − .229, SE = .025, *p *< .001, 90% CI [− .091, − .016]). Meanwhile, when examined separately, two indirect paths were significant: psychological *suzhi *→ self-esteem → social anxiety (^*®*^= − .095, SE = .027, *p *< .001, 90% CI [− .047, − .018]) and psychological *suzhi *→ self-esteem → sense of security → social anxiety (^*®*^ = − .151, SE = .018, *p* < .001, 90% CI [− .061, − .039]). However, the mediating effects of sense of security on the relationship between psychological *suzhi* and social anxiety were not significant. Consequently, the total indirect effect of self-esteem and sense of security on the relationship between psychological *suzhi* and social anxiety was .656. In addition, self-esteem was found to mediate the relationship between psychological *suzhi* and sense of security (^*®*^= .381, SE = .031, *p *< .001, 90% CI [.350, .474]), and sense of security was found to mediate the relationship between self-esteem and social anxiety (^*®*^= − .256, SE = .027, *p *< .001, 90% CI [− .117, − .081]).

**Table 2:** Standardised indirect effects of psychological suzhi on social anxiety

Indirect effect	^*®*^	*SE*	*p*	90% CI
*Suzhi *→ SA	− .229	.025	.000	− .091, − .016
*Suzhi *→ SS → SA	− .394 × .046 = .018	.016	.025	− .003, .014
*Suzhi *→ SE → SA	− .194 × .438 = − .095 − .091	.027	.000	− .047, − .018
*Suzhi *→ SE → SS → SA	− .39 × .586 × .649 = − .151	.018	.000	− .061, − .039

*Suzhi* = psychological *suzhi*, SE = self-esteem, SS = sense of security, SA = social anxiety

## Discussion

This study analysed the effects of psychological *suzhi* on social anxiety and extended the literature by investigating the potential mediating effects of self-esteem and sense of security in this relationship. Consistent with Hypothesis 1, we discovered that psychological *suzhi* is positively related to self-esteem and sense of security, and it is negatively related to social anxiety.

The finding that higher psychological *suzhi* predicts lower social anxiety is consistent with the results of previous research conducted with Chinese adolescents [23], and it indicated that adolescents’ psychological *suzhi* is an important protective factor for social anxiety. There are several possible explanations for this finding. First, the diathesis-stress model suggests that certain underlying vulnerabilities combined with stressful life events result in the development of mental disorders, whereas protective factors serve to mitigate the impact of stressful life events [42]. In this regard, as a positive psychological quality, psychological *suzhi* can effectively help teenagers relieve the pressure they experience during social interactions in their daily lives, which results in fewer psychological problems like anxiety and depression. Second, psychological *suzhi* predicts good peer relationships, as middle school students with high psychological *suzhi* can more effectively cope with stressful events because of their improved interpersonal communication skills and ability to adapt to various types of social environments [43]. Having these positive peer interactions can, in turn, prevent and alleviate social anxiety. This finding corroborates those of studies examining the association between psychological *suzhi* and mental health, which have found that psychological *suzhi* positively relates to mental health [44]. Thus, the current finding adds empirical support for the relationship model of psychological *suzhi* and mental health [45].

Further, the finding that psychological *suzhi* is positively correlated with self-esteem is also consistent with previous research [7, 23]. Psychological *suzhi* concerns a unification of the content of individual psychological and behavioural factors (cognitive, personality) with functional value (adaptive); thus, it constitutes the inner basis for the formation of various psychological functions and the improvement in behavioural efficiency. Moreover, it can improve individuals’ health and foster the development of more adaptive personality traits. Psychological s*uzhi* is also the foundation for middle school students’ success across various settings (e.g. academic, interpersonal) and forms the basis for their realisation of life values. Meanwhile, self-esteem refers to individuals’ positive self-evaluations and positive emotional experiences within social contexts [7]. Therefore, psychological *suzhi* is an important catalyst for students’ self-esteem, and high psychological quality predicts high self-esteem.

Finally, the finding that psychological *suzhi* positively relates to sense of security also supports our hypothesis. One possible explanation for this result is that psychological *suzhi* is an endogenous factor of mental health [45], and sense of security is one of nine main mental health criteria [46]. Thus, psychological *suzhi* may predict a sense of security.

Hypothesis 2 was also supported in this study, as self-esteem and sense of security were found to play a mediating role in the relationship between psychological *suzhi* and social anxiety, with the mediating effect equalling 65.6%. Psychological *suzhi* was determined to have a direct effect on social anxiety and an indirect effect on it through self-esteem and sense of security. Specifically, self-esteem and sense of security mediated the relationship between psychological *suzhi* and social anxiety through two significant paths. The first of these was psychological *suzhi *→ self-esteem → social anxiety, which had an effect of 27.2%. This result suggests that psychological *suzhi* is an important catalyst for students’ self-esteem. Individuals with high self-esteem tend to have more positive views of themselves and, in the process of interacting with people, show more initiative. However, those with low self-esteem have a more negative self-evaluation, and they are more passive in their interpersonal communication [47]. As a result, adolescents with low self-esteem have greater social anxiety. This finding is consistent with the cognitive behavioural theory of social anxiety, which suggests that low self-esteem is the main cause of social anxiety [24]. The second path between psychological *suzhi* and social anxiety was psychological *suzhi *→ self-esteem → sense of security → social anxiety, for which the mediating effect was 43.3%. This finding indicates that self-esteem and sense of security serve as chain mediators in the relationship between psychological *suzhi* and social anxiety. Indeed, past research has found that sense of security is associated with self-esteem. For example, Klandermans and van Vuuren [48] found that certain personality characteristics, such as self-esteem, determine perceptions of job insecurity. Similarly, Kinnunen et al. [49] showed that low self-esteem can significantly predict subsequent job insecurity. Further, the sociometer theory of self-esteem proposed that people with high self-esteem have a sense of competence and value. They are able to handle problems associated with social interactions and higher security and control; consequently, they have less interpersonal anxiety [50]. With regard to the finding that sense of security is negatively related to social anxiety, past studies have revealed that sense of security is positively associated with interpersonal relationships, and successful interpersonal interactions help individuals form a high level of self-esteem in social situations and alleviate social anxiety [51]. Therefore, self-esteem may influence individuals’ sense of security, which, in turn, affects their social anxiety. Thus, sense of security served to mediate the relationship between self-esteem and social anxiety, psychological *suzhi* promotes self-esteem, and self-esteem and sense of security serve as chain mediators in the relationship between psychological *suzhi* and social anxiety. This finding implies that, as a comprehensive psychological construct, psychological *suzhi* can influence individuals’ self-evaluations and their perceptions and control of interpersonal security. It can also predict the occurrence of social anxiety. The discovery of this mediating role will help reduce social anxiety by starting with self-esteem and sense of security.

As with any study, this current one has some limitations. First, this study was cross-sectional in nature, which precludes any causal inferences. Thus, future longitudinal or experimental research is needed to identify the possible causal relationships. Second, only Chinese adolescent students were included; consequently, caution is needed when generalising these results to other cultures or age groups. Despite these shortcomings, this study still has great theoretical and practical significance. In particular, the present study has important implications for the theoretical construction and practical treatment of social anxiety. Theoretically, the findings demonstrate a new function of psychological *suzhi* based on its influence on social anxiety via self-esteem and sense of security, which is consistent with the relationship model of psychological *suzhi* and mental health [45]. Future research is needed to further examine the role that sense of security plays in preventing social anxiety and protecting mental health, given its relationship with psychological *suzhi,* as revealed in the current study. Moreover, the current findings have several practical implications. First, school leaders and psychology teachers should plan and implement routine psychological *suzhi* training courses to improve students’ psychological *suzhi*, thereby preventing social anxiety. Second, when school counsellors interview students with low psychological *suzhi* and high levels of social anxiety, they can support these students by encouraging them to participate in extracurricular activities and gain positive self-experience from these activities. Doing so can help these students develop positive self-perceptions [52], consequently helping them develop basic interpersonal communication skills and alleviating their senses of insecurity and uncertainty.

## Conclusions

As anticipated, psychological *suzhi*, self-esteem, sense of security, and social anxiety were closely related to each other. Moreover, self-esteem and sense of security were determined to mediate the relationship between psychological *suzhi* and social anxiety. Notably, the chain mediating effect of self-esteem and sense of security was very strong. This result implied that psychological *suzhi* can directly protect adolescents from social anxiety, and it can also protect them by increasing their self-esteem and sense of security. The results of this study provide a new perspective for the prevention and treatment of social anxiety. In addition, they hold great implications for the prevention and treatment of social anxiety within a campus environment. It is important for education agencies and families to reinforce adolescents’ psychological *suzhi* in various ways, including training in psychological *suzhi* and its components. These findings are also of great significance to the practical work of psychological counselling.

## Abbreviations

*Suzhi*: psychological *suzhi*
SE: self-esteem
SS: sense of security
SA: social anxiety
